# Editorial: Risk Factors and Outcome Predicating Biomarker of Neurodegenerative Diseases

**DOI:** 10.3389/fneur.2019.00045

**Published:** 2019-02-04

**Authors:** Chaur-Jong Hu, Jean-Noël Octave

**Affiliations:** ^1^Department of Neurology, Shuang Ho Hospital, Taipei Medical University, New Taipei City, Taiwan; ^2^Department of Neurology, School of Medicine, College of Medicine, Taipei Medical University, Taipei, Taiwan; ^3^The Ph.D. Program for Neural Regenerative Medicine, College of Medical Science and Technology, Taipei Medical University, Taipei, Taiwan; ^4^Taipei Neuroscience Institute, Taipei Medical University, New Taipei City, Taiwan; ^5^Institute of Neuroscience, Catholic University of Louvain, Louvain-la-Neuve, Belgium

**Keywords:** neurodegeneration, Alzheimer's disease, Parkinson's disease, predication, biomarker

Neurodegenerative diseases, such as Alzheimer's disease (AD), Parkinson's disease (PD), and amyotrophic lateral sclerosis (ALS) are major public health issues in the aging population. The central nervous system undergoes considerable degeneration for years before the onset of cardinal neurodegenerative symptoms such as resting tremors in PD (1). Failure to identify highly disease-correlated risk factors or biomarkers delays the initiation of neuroprotective treatment; several clinical trials on disease modification in neurodegenerative disorders have failed because of this. The lack of reliable outcome-predicting biomarkers hinders the development of disease modification approaches (2). Epidemiological studies have presented significant risk factors for and biomarkers of neurodegenerative diseases that can be detected before diagnosis, such as mild cognitive impairment (MCI) for AD (3, 4) and rapid eye movement sleep behavioral disorder (RBD) for PD (5). Vascular risk factors accelerate AD progression (6); diabetes is associated with rapid deterioration in PD (7). However, this knowledge does not facilitate robust prediction of neurodegenerative diseases and outcomes or early initiation of neuroprotective treatment in clinical trials.

In this special issue, we have gathered eight original articles regarding biomarkers of neurodegenerative diseases. A review article explores the role of neuroimaging in neurodegenerative diseases. Articles in this special issue address three topics: clinical disease biomarkers for neurodegenerative diseases, biomarkers from animal models of neurodegenerative diseases, and biomarkers as therapeutic indicators.

## Clinical Disease Biomarkers For Neurodegenerative Diseases

Dolatshahi et al. used the database of the Parkinson's Progression Markers Initiative project for analyzing the levels of alpha-synuclein (α-syn), total tau (t-tau), phosphorylated tau (p-tau), and beta-amyloid (Aβ1–42) in cerebrospinal fluid (CSF) to determine associations between the levels of each protein and baseline clinical manifestations; Aβ1–42 levels did not differ significantly between PD and control cohorts at any time; t-tau and α-syn levels differed significantly. Only the baseline RBD questionnaire scores were predictive of temporal alterations in the α-syn levels. Longitudinal changes in the levels of CSF proteins are mutually related, and RBD is a promising prognostic predictor of PD progression. Joo et al. investigated body mass index (BMI) and the risk of progression from MCI to AD, and effects of BMI on progression to AD with age, sex, cognitive intervention, and chronic diseases as variables. Clinical data obtained from 388 patients with MCI over 36.3 ± 18.4 months of follow-up indicated that the underweight patients with MCI had a relatively high risk of AD conversion. Subgroup analysis showed that this effect was strongly observed among patients who were female, at least ≥75 years old, did not receive cognitive intervention, and had hypertension. Being underweight was a potential biomarker for a high MCI risk. Cho et al. investigated baseline serum vitamin B12 levels and the progression of cognitive impairment in patients with AD who received cholinesterase inhibitors (ChEI). In 165 Taiwanese patients with mild to moderate AD who received ChEI for at least 2 years, the rates of cognitive decline were significantly lower in the patients with optimal (above the median level) than in those with suboptimal baseline vitamin B12 levels. After adjusting for several variables, the suboptimal baseline serum vitamin B12 level was a significant predictor of cognitive decline. Hsieh et al. retrospectively reviewed the medical records of patients with sporadic Creutzfeldt–Jacob disease (sCJD) to identify predictive variables for early initiation of artificial feeding, which indicates swallowing failure; the presence of myoclonus in the early stages and the elevated CSF levels of 14-3-3 protein were associated with early artificial feeding, which may indicate rapid deterioration in sCJD. Stanga et al. analyzed amyloid precursor protein (APP) processing by measuring the levels of its soluble nonamyloidogenic fragments (sAPPα and sAPPβ), amyloid-β (Aβ peptides), and glial cell line–derived neurotrophic factor (GDNF) levels in the CSF and serum samples obtained from patients with ALS and healthy controls; alterations in GDNF and sAPPα levels in the samples were associated with a moderate and rapid progression of ALS. GDNF levels were markedly lower in the serum of the patients with ALS, while sAPPα levels were higher than those in the healthy controls. Shimizu et al. reviewed the role of neuroimaging for biomarkers of neurodegenerative diseases. This review describes magnetic resonance imaging, single photon emission computed tomography, positron emission tomography, amyloid imaging, dopamine transporter imaging, and 123I-metaiodobenzyl-guanidine myocardial scintigraphy, for biomarkers of AD, PD, and progressive supranuclear palsy.

## Biomarkers From the Animal Models of Neurodegenerative Diseases

Huang et al. considered circadian rhythm dysfunction (CRD) and the progression of ALS in SOD1G93A ALS model mice; cCRD accelerated ALS onset and progression and increased the loss of motor neurons, activated gliosis, and nuclear factor κB-mediated inflammation in the spinal cord. CRD also resulted in an increase in the abundance of enteric cyanobacteria in the ALS model mice, which may be involved in the pathogenesis of ALS. Lu et al. used 1-Methyl-4-phenyl-1,2,3,6-tetrahydropyridine (MPTP)-induced PD mice to explore the cerebral metabolic bases of the occurrence and development of PD. The alteration of metabolite levels in the striatum and substantia nigra of the mice on days 1, 7, and 21 after MPTP injection were assessed through ^1^H nuclear magnetic resonance spectroscopy; in the striatum, the glutamate levels increased significantly and persistently after PD induction; N-acetylaspartate levels increased transiently after PD induction but soon declined and were identical with the controls. Key enzymes in the glutamate–glutamine cycle increased significantly in the PD mice. Stanga et al. considered animal models of ALS; the results indicated that both GDNF and APP levels increased in the hind limb muscles of the transgenic ALS mouse models at the onset of motor deficits.

## Biomarkers as Therapeutic Indicators

Oxidative stress is a major pathogenic factor of neurodegenerative diseases; oxidative stress markers can be used to evaluate the therapeutic effect of antioxidants. Al-Amin et al. investigated the antioxidative effects of risperidone on lipopolysaccharide (LPS)-induced oxidative stress in mice; risperidone significantly reduced the LPS-induced increase in the levels of the lipid peroxidation product malondialdehyde, advanced protein oxidation products, and nitric oxide in the cortex. Risperidone attenuated LPS-induced neuronal oxidative damage in the cortex through its ability to reduce reactive oxidative stress.

Biomarkers are essential for all types of neurodegenerative diseases. Determination of either clinical or experimental biomarkers provides additional knowledge about disease mechanisms, outcome prediction, and selection of therapeutic targets.

## Author Contributions

All authors listed have made a substantial, direct and intellectual contribution to the work, and approved it for publication.

### Conflict of Interest Statement

The authors declare that the research was conducted in the absence of any commercial or financial relationships that could be construed as a potential conflict of interest.
